# 1846. Outcomes in Intravenous to Oral Antimicrobial Therapy in Beta-Streptococcus Species

**DOI:** 10.1093/ofid/ofac492.1475

**Published:** 2022-12-15

**Authors:** Mackenzie R Keintz, Cristina J Torres, Molly M Miller, Bryan T Alexander, Elizabeth Lyden, Elizabeth Lyden, Elizabeth Lyden, Jihyun Ma, Trevor C Van Schooneveld, Jasmine R Marcelin

**Affiliations:** University of Nebraska Medical Center, Omaha, Nebraska; University of Nebraska Medical Center, Omaha, Nebraska; Nebraska Medicine, Omaha, Nebraska; Nebraska Medicine, Omaha, Nebraska; University of Nebraska Medical Center, Omaha, Nebraska; University of Nebraska Medical Center, Omaha, Nebraska; University of Nebraska Medical Center, Omaha, Nebraska; University of Nebraska Medical Center, Omaha, Nebraska; University of Nebraska Medical Center, Omaha, Nebraska; University of Nebraska Medical Center, Omaha, Nebraska

## Abstract

**Background:**

Uncomplicated bloodstream infections (uBSI) are common and often receive prolonged courses of intravenous (IV) antibiotics, increasing risk for catheter-associated complications and hospitalization costs. β-hemolytic Streptococcus spp. are a common cause of BSI and have reliable susceptibility to many oral antibiotics. Clinically improving patients without persistent BSI and a controlled source of infection are candidates for oral antimicrobial therapy (OAT) but despite anecdotal practice, there are few studies affirming the practice of OAT for gram-positive uBSI. We evaluated IV to OAT transitions for treating β-hemolytic streptococcal uBSI.

**Methods:**

This retrospective cohort study included patients >18 years old hospitalized between 1/1/2013 and 12/31/2019 diagnosed with uBSI due to β-hemolytic Streptococcus. Patients were excluded if BSI was due to endovascular, central nervous, or bone/joint infection without source control. We compared outcomes in patients treated with IV only to those transitioned to OAT including: 30-day mortality, antimicrobial therapy, BSI relapse, 30-day rehospitalization, adverse drug events, and reversion to IV therapy. Fisher’s exact test was used for categorical variables; Mann-Whitney test and independent t-test for continuous variables.

**Results:**

A total of 238 Streptococcus BSI (of 321 BSI screened) were included (83 excluded as complicated, pediatric, or outpatient). OAT was used in 153 (64%). Cohort demographics were similar (table 1). Infectious disease (ID) consultation was not statistically associated with OAT transition; in fact, ID consults tended to use less OAT (66% IV vs. 54% OAT p=0.10). Hospital length of stay was statistically shortened in the OAT cohort with a median of 5 (interquartile range 4) vs. 7.5 (10.5) (p< 0.0001). Patients transitioned to OAT were more likely to finish their antibiotic course outpatient (93 vs. 61% p< 0.001). Thirty-day mortality was decreased in the OAT cohort (2% vs. 13% p< 0.0001). Adverse events were not statistically significant between the groups.

**Figure:**
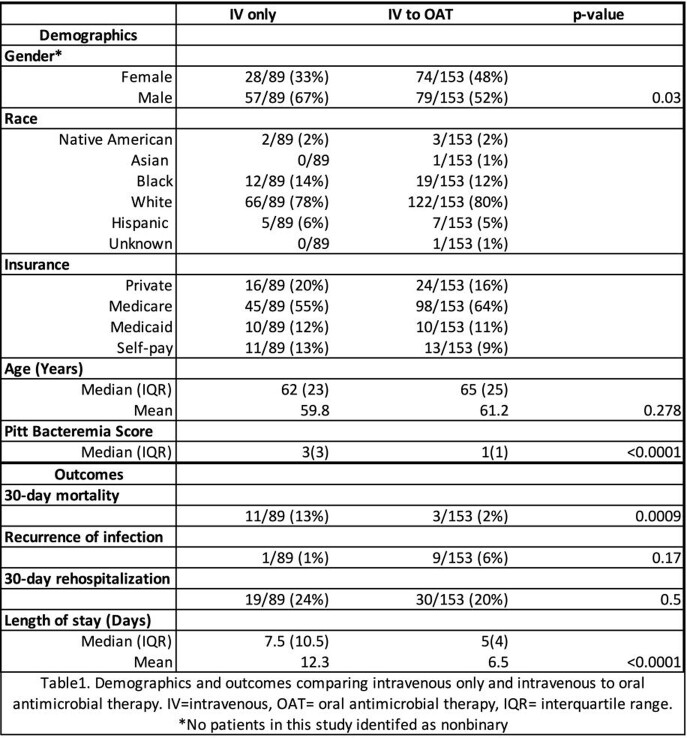

**Figure:**
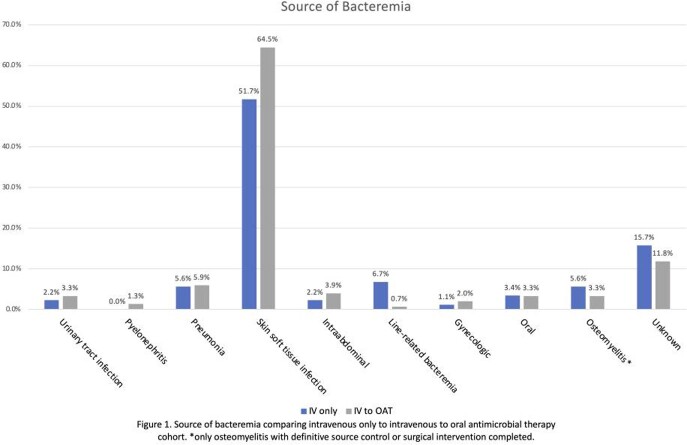

**Conclusion:**

Opportunities exist to modify practice management of uBSI. For β-hemolytic Streptococcus uBSI, OAT was associated with decreased length of stay without adverse clinical outcomes.

**Disclosures:**

**Bryan T. Alexander, PharmD, BCIDP, AAHIVP**, Astellas Pharma: Advisor/Consultant **Trevor C. Van Schooneveld, MD**, bioMerieux: Advisor/Consultant|bioMerieux: Grant/Research Support|Insmed: Grant/Research Support|Merck: Grant/Research Support|Thermo-Fischer: Advisor/Consultant **Jasmine R. Marcelin, MD**, Pfizer (Grant reviewer): Honoraria.

